# Comparative analysis of the leprosy detection rate regarding its clinical spectrum through PCR using the *16S rRNA* gene: a scientometrics and meta-analysis

**DOI:** 10.3389/fmicb.2024.1497319

**Published:** 2024-10-21

**Authors:** Marcos Jessé Abrahão Silva, Thiago Pinto Brasil, Caroliny Soares Silva, Cristiane Cunha Frota, Daniele Melo Sardinha, Luiza Raquel Tapajós Figueira, Keitty Anne Silva Neves, Everaldina Cordeiro dos Santos, Karla Valéria Batista Lima, Nédia de Castilhos Ghisi, Luana Nepomuceno Gondim Costa Lima

**Affiliations:** ^1^Ph.D and Master Program in Parasitic Biology in the Amazon (PPGBPA), State University of Pará (UEPA), Belém, Pará, Brazil; ^2^Bacteriology and Mycology Section (SABMI), Evandro Chagas Institute (IEC), Ananindeua, Pará, Brazil; ^3^Universidade Tecnológica Federal do Paraná (UTFPR), Dois Vizinhos, Paraná, Brazil; ^4^Faculty of Medicine, Federal University of Ceará (UFC), Fortaleza, Ceará, Brazil; ^5^Programa de Pós-Graduação em Biotecnologia, Universidade Tecnológica Federal do Paraná (UTFPR), Dois Vizinhos, Paraná, Brazil

**Keywords:** paucibacillary leprosy, multibacillary leprosy, diagnosis, polymerase chain reaction, *Mycobacterium leprae*

## Abstract

**Background:**

Leprosy is a chronic and disabling infectious disease caused by *Mycobacterium leprae*. It has a wide clinical spectrum and is operationally classified into paucibacillary (PB) and multibacillary (MB) cases. There is evidence that the *16S rRNA* gene can be used in Polymerase Chain Reaction (PCR) for complementary detection with high sensitivity and specificity. However, there is no literature convention on its diagnostic correspondence regarding the particular operational classification of the disease. This study aimed to correlate, through a meta-analysis, the detection rate of leprosy between the PCR method with the *16S rRNA* gene in the clinical forms PB and MB in relation to confirmed cases.

**Methods:**

This is a systematic review and meta-analysis study conducted according to the PRISMA 2020 guidelines, using the search descriptors with “AND”: “Leprosy”; “Polymerase Chain Reaction”; “*16S rRNA*” in the PUBMED, SciELO, LILACS, and Science Direct databases. The search was limited to original observational articles in Portuguese, English, or Spanish, with no defined time frame. The methodological quality assessment of the selected articles was performed using the JBI checklists. A scientometric approach to the article using used the VOS Viewer and Scimago Graphica software. The meta-analysis was conducted using Comprehensive Meta-Analyses software, under Pearson’s Correlation effect test and fixed effect model and subgroup analysis concerning the type of sample analyzed.

**Results:**

The study was significant from the perspective of the paucibacillary group (Clinical biopsy: -0.45 [95% CI= -0.63 – −0.22], *p* < 0.001/ Slit smear skin: −0.52 [95% CI= -0.65 – −0.36], *p* < 0.001 / Overall: −0.50 [95% CI= −0.61 – −0.37], *p* < 0.001). The PCR diagnostic method for the16S rRNAgene ofM. lepraehas low viability and diagnostic sensitivity in both clinical biopsy samples and leprosy skin smears.

**Conclusion:**

This implies little validation of it as a PCR target gene for diagnosing the disease, highlighting limitations in the actual technique.

**Systematic review registration:**

https://www.crd.york.ac.uk/prospero/, identifier CRD42024588790.

## Introduction

Leprosy, caused by *Mycobacterium leprae* and *M. lepromatosis*, is a chronic disease that affects the skin and peripheral nerves, resulting in physical disabilities and severe deformities. This significantly impacts the mental health and quality of life of those affected (Ebenezer and Scollard, 2021; Santos et al., 2020). With cases documented for millennia, it is considered one of the oldest diseases (Mottaghi et al., 2020). Those affected by this infection were segregated and stigmatized by historical society due to several factors: lack of effective treatments, disfigurement caused by the disease, high infectivity, belief in divine punishment, and fear of transmission (Sil and Das, 2022).

Several aspects of leprosy have already been elucidated by science. However, there are gaps in leprosy diagnosis that include issues of sensitivity and specificity, difficulties in differentiating between active and latent infections, and the need for a better understanding of the diagnostic yield in different clinical forms of leprosy, specifically between paucibacillary (PB) and multibacillary (MB) cases. Research indicates that PB cases often show lower PCR positivity than MB cases due to their inherently lower bacterial loads (Martinez et al., 2014). In the context of leprosy, this load varies significantly between different clinical forms of the disease. MB cases typically have a higher bacterial load compared to paucibacillary PB cases. This difference directly impacts the sensitivity of PCR assays; higher bacterial loads increase the likelihood that sufficient target DNA will be present for amplification (Martinez et al., 2006; Truman et al., 2008).

There is a pressing need for studies that explore the specific conditions under which PCR can be optimized for detecting *M. leprae* DNA in PB cases. Understanding how different types of samples and PCR techniques affect diagnostic yield could improve early detection strategies for these patients, and research into new target markers and diagnostic techniques could reduce under-detection caused by a possible low level of bacterial load in some specimens (Reis et al., 2014).

Although there is treatment for the disease through multiple drug therapy (which has significantly reduced its prevalence), it occurs endemically in 105 countries located in Southeast Asia, the Americas, Africa, the Eastern Pacific, and the Western Mediterranean, which exhibit high case rates (Alrehaili, 2023). Transmission occurs, regardless of sex or age, due to direct contact between the patient and predisposed individuals, probably through inhalation of droplets from the upper respiratory tract (Guevara et al., 2022). Socioeconomic disparities contribute to ongoing transmission. For example, many affected individuals in endemic areas of Brazil come from impoverished backgrounds where access to healthcare is limited, leading to delays in diagnosis and treatment. This situation perpetuates stigma and discrimination against those affected by leprosy, further complicating public health efforts (Santos et al., 2024).

On a global scale, the highest occurrence rates for the disease are recorded in adults; however, a significant proportion is also observed in people under the age of 15 (Castillo et al., 2021). The World Health Organization (WHO) recorded around 174,087 new cases of leprosy worldwide in 2022, of which 21,387 occurred in the Americas (Organização Mundial da Saúde, 2024). With Brazil registering 19,635 new cases, the country ranks first in the American continent and second in the world in terms of the incidence rate of new cases of leprosy in the year 2022 (Ministério da Saúde, 2024; Mártires et al., 2024).

Brazil reports over 30,000 new leprosy cases annually, making it one of the countries with the highest burden of the disease globally. Despite a general decline in new case detection rates (NCDR), certain regions, with more evident socio-economic inequalities, especially Pará State, Northern Brazil, continue to experience high incidence rates, with NCDR reaching 25.7 per 100,000 population in 2017 (Dergan et al., 2023). Southeast Asia has the highest burden of leprosy among WHO regions, accounting for about 67% of global new cases. Countries like India, Bangladesh, and Nepal are particularly affected, with significant populations at risk due to poor living conditions and limited healthcare access (Wang et al., 2023).

The clinical spectrum of leprosy depends on the individual’s immune response to *M. leprae*. The Ridley-Jopling division, which highlights the variety of host responses, categorizes patients (Lockwood et al., 2022). This typology stratifies cases into two polar forms: tuberculoid leprosy (TT) and lepromatous leprosy (LL). In addition to these polar forms, there are other clinical classifications, including borderline tuberculoid (BT), mid-borderline (BB), and borderline lepromatous (BL) (Degechisa and Dabi, 2022).

However, to define treatment, the WHO defined an easy classification, in which leprosy patients can be divided into two categories depending on the number of skin lesions and the bacillary rate, when this is accessible, in paucibacillary (PB) (up to five lesions) or multibacillary (MB) (more than five lesions) (Beissner et al., 2019; Germano et al., 2022). Here, it is important to highlight that diagnosing in the early stages of leprosy is essential to establish effective control of the disease (Rusmawardiana et al., 2022). Bacilloscopic, serological, and histological tests are some laboratory tests that are applied in the investigation of leprosy. However, no exam performed in isolation can diagnose the disease, as results may vary between methods according to the clinical types of the disease (Torres et al., 2021).

Confirming the diagnosis requires an analysis that considers the patient’s clinical history, anamnesis, laboratory evidence, and investigation of peripheral nerve injuries (Kundakci and Erdem, 2019). However, some molecular testing techniques have been evaluated to develop tests with greater performance, such as the polymerase chain reaction (PCR), which has a high sensitivity and specificity rate for detecting *M. leprae* DNA (Gama et al., 2020). Over the past two decades, several PCR methods have been developed for the amplification of a variety of *M. leprae* gene targets, including the 36 kDa antigen, the 18 kDa antigen, the 65 kDa antigen, and the *16S rRNA* (Chen et al., 2019).

Ribosomal RNA molecules are of particular taxonomic interest, especially the *16S rRNA* gene (Turankar et al., 2015). The *16S rRNA* gene encodes the 16S part of ribosomal RNA, which is essential for protein synthesis and ribosomal function in all bacteria. It is well conserved among bacterial species despite being particular to each of them in its genetic constitution. In common with other slow-growing mycobacteria, *M. leprae* has a single copy of the gene *rRNA* with a large part of the recognized sequence (95’i) in the *16S rRNA* gene, indicating a close relationship between *M. leprae*, *M. tuberculosis* and *M. avium* (Teske et al., 1991).

Ribosomes are RNA-based cellular components that function in protein synthesis. Species-specific insertions and deletions can be observed in distinct regions of rRNAs (ribosomal RNAs) (Kushwaha and Bhushan, 2020). In the case of the *M. leprae 16S rRNA* gene, a unique short sequence of 16 base pairs (bp) rich in adenine (A) and thymine (T) is observed. In contrast, in *M. lepromatosis,* one can find a sequence with a high proportion of AT composed of 19 bp included in its *16S rRNA* gene (Deps and Collin, 2021).

The disease presents a significant public health challenge, particularly in endemic regions. Traditional diagnostic methods, including clinical assessments and histopathological examinations, often need more sensitivity, especially in paucibacillary cases (Sharma et al., 2024). The advent of molecular techniques, particularly PCR targeting the *16S rRNA* gene, has revolutionized the detection of *M. leprae* DNA, offering a more sensitive and specific alternative.

Furthermore, immunological considerations are crucial in understanding leprosy, as the clinical spectrum of the disease is closely tied to the host’s immune response. The interplay between *M. leprae* and the immune system determines whether an individual develops a PB or MB form of leprosy. The immune response to *M. leprae* is characterized by a T helper (Th)1/Th2 paradigm. PB leprosy is associated with a robust Th1 response, marked by high levels of pro-inflammatory cytokines such as interferon-gamma (IFN-*γ*), facilitating effective cell-mediated immunity. In contrast, MB leprosy typically presents a Th2-dominated response with elevated levels of interleukin (IL)-4 and IL-10, leading to a humoral immune response and higher bacillary loads (Silva et al., 2024). Molecular diagnostics, particularly PCR, can complement immune-based diagnostic approaches, providing a more comprehensive view of the disease. This can be exemplified by the fact that PCR offers superior sensitivity for detecting *M. leprae* DNA in PB patients who may not exhibit significant antibody responses or visible bacilli on microscopy (Tatipally et al., 2018; Gama et al., 2020).

The choice of the *16S rRNA* gene as a target for PCR in leprosy diagnostics is based on several important factors that highlight its relevance and utility. The *16S rRNA* gene is highly conserved across bacterial species, but specific sequences within this gene are unique to *Mycobacterium leprae*. This specificity ensures that PCR assays targeting the *16S rRNA* gene can reliably identify the presence of *M. leprae* without cross-reactivity with other mycobacterial species, making it a suitable choice for leprosy diagnosis (Teske et al., 1991). The use of *16S rRNA* in PCR assays is well-documented and established in clinical microbiology. PCR targeting the *16S rRNA* gene can be applied to various clinical samples, including skin biopsies, nasal swabs, and blood. While primarily used for detecting DNA, modifications of the assay (e.g., reverse transcription PCR [RT-PCR]) can enable the detection of RNA, providing insights into the viability of *M. leprae*. This capability can help differentiate between active infections and latent or non-viable organisms (Beissner et al., 2019).

The purpose of the present study was to compare the detection rates of leprosy in PB and MB patients using PCR targeting the *16S rRNA* gene. For this reason, a meta-analysis was conducted here in order to synthesize data from multiple studies and provide a more comprehensive understanding of the diagnostic efficacy of PCR using *16S rRNA*.

## Materials and methods

### Study design

This is a systematic review and diagnostic accuracy meta-analysis, based on the PRISMA (Preferred Reporting Items for Systematic Reviews and Meta-Analyses) 2020 writing protocol to achieve the proposed objective of the study, and with scientometrics analysis, based on Preferred Reporting Items for Systematic reviews and Meta-Analyses extension for Scoping Reviews (PRISMA-ScR) (Page et al., 2021). This review was registered in PROSPERO with code CRD42024588790.

### Scientometrics analyzes

The inclusion criteria for articles for scientometrics were to be consistent with the theme of molecular biology; PCR; diagnosis; *16S rRNA*; Leprosy. The program Vosviewer 1.6.6 (Leiden University, Leiden, The Netherlands) was used to analyze the collaboration between authors, and the main keywords involved in this manuscript. Furthermore, Scimago Graphica (Version 1.0.17, Scimago, Granada, Spain) was used to construct the map of the level of publication on the topic with the references used in this article over the years^1^ (accessed August 14, 2024).

### Formulation of the guiding question

To formulate the guiding question of this meta-analysis, the PICO (Population; Intervention; Comparison; Outcome) strategy was used and consisted of the following anagram: (P): patients with leprosy; (I): compare the leprosy detection rate by PCR with *16S rRNA* regarding the clinical spectrum; (C): cases diagnosed in each operational classification in relation to real cases; (O): detection rate generated (Santos et al., 2007). This perspective refers to comparing clinical diagnosis versus molecular diagnosis, considering that the real cases were those diagnosed clinically (gold standard method).

### Literature search strategy and eligibility criteria

The keywords (DeCS/MeSH) used for the search were: “*16S rRNA*”; “Leprosy”; “Polymerase Chain Reaction,” together with “AND”. The databases investigated were: PUBMED, SciELO, Science Direct and LILACS. Studies in English, Spanish, or Portuguese were searched for the entire time frame available in them. For inclusion of the study types, observational case–control, cross-sectional, and cohort studies were eligible. Brief/short communications, letters to the editor, editorials, articles available in abstract format, and articles unavailable in their full form were excluded.

### Data extraction

The search in the databases, collection, investigation, tabulation, and data extraction was carried out by two authors independently (MJAS and TPB). They organized with the help of Microsoft Office Excel 365 software. Any disagreement between the analyses was resolved with the help of a third researcher (CSS). The data was extracted in July, 2024. The data extracted from the articles were: authors, year of publication, title, source database, methodology, sampling, nature of the sample, location of the population, and results of detection rate.

### Assessment of the methodological quality of the articles and data synthesis

The quality assessment was performed by two researchers (MJAS and TPB) using the Joanna Briggs Institute - JBI critical appraisal checklist for analytical cross-sectional studies (score 0–8), the JBI checklist for case–control studies (score 0–10), and the JBI checklist for cohort studies (score 0–11). Only when the conditional answer was “Yes” will the scores for completing the checklist questions be considered. A third researcher gave an opinion in case of disagreement (CSS) (Aromataris and Munn, 2017). The evaluations were carried out, and the inclusion criterion for the studies as high quality in terms of methodological quality was if they obtained more than 60% of the corresponding scores (Silva et al., 2023). The synthesis of the generated data was done in tabular form and through the forest plot.

### Statistical analysis of the meta-analysis

The Comprehensive Meta-Analyses–CMA program, version 2.2 (Biostat, Englewood, NJ, USA), was used on a computer to perform the statistical analysis of the meta-analysis. The effect test used was Pearson’s correlation to assess the connection and stability of the results across the operational classification groups of leprosy. The fixed-effects model estimated the frequency rate relationships in a combined manner with 95% confidence intervals (95% CI). A subgroup analysis was performed concerning the nature of the sample (samples from skin smears or biopsies). The Cochrane *Q* test and the *I*-squared measure (*I*^2^) were used to determine heterogeneity between the groups (*p* < 0.05 was considered statistically significant) (Sharpe, 2015). The Begg’s rank correlation test and a funnel plot were used to examine the potential for publication bias (*p* < 0.05 will be considered statistically significant). Subgroup analyses were done using the types of samples used for molecular testing in each study.

## Results

### Literature search

**Figure 1:**
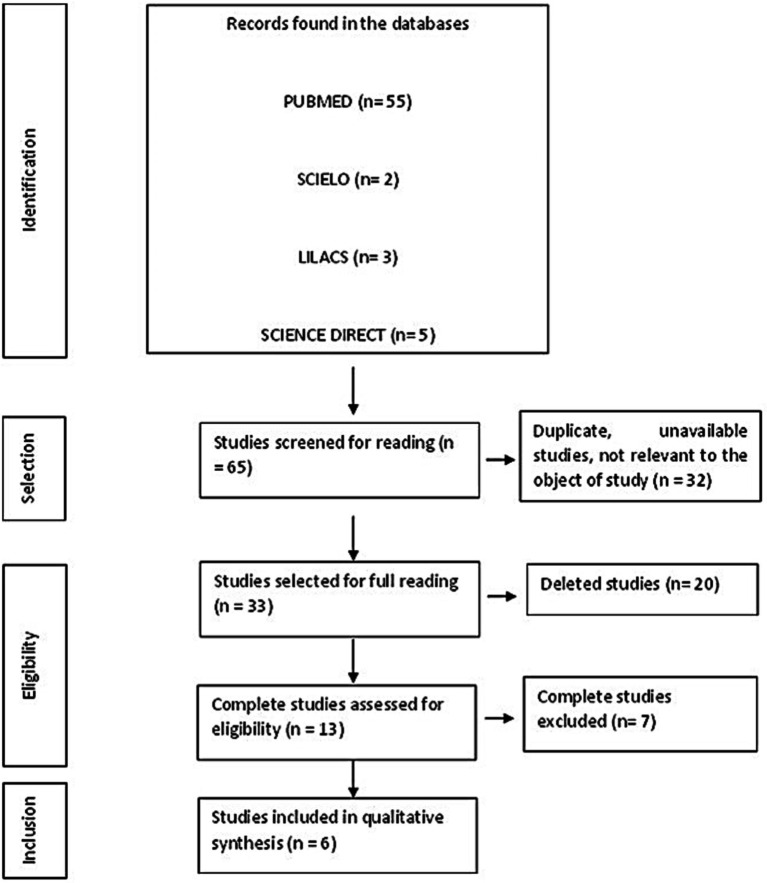
PRISMA flowchart for eligibility and inclusion of studies in this review.

Out of the 65 papers that were initially found, 24 studies were removed throughout the data selection process. These studies included 8 letters to the editor, 7 studies that were only available in abstract form, and 9 duplicates. In addition, eight papers were deemed irrelevant to the subject based on an assessment of the title, abstract, and content. The authors separately discarded an additional 27 studies after analyzing the complete text of each publication and applying the qualifying criteria. The final dataset from this method is shown in this review and is further explained in Figure 1.

### Characterization of included studies

The six studies included in this meta-analysis were all from PUBMED (100%), in English (100%), with most Asian populations (50%), of which 33.34% were Indian and 16.66% Thai, followed by American (Brazilian) with 33.34% and African (Ethiopian) 16.66%. Furthermore, the samples used as an experimental basis in these studies were methodologically based on cross-sectional studies in half of them and case–control studies in the other half. Regarding the nature of the samples in these studies, 50% were from skin smears and the other 50% were from clinical biopsies. The methodological quality assessment of these studies considered the quality to be high (Table 1).

**Table 1:** Characteristics of the studies included in this meta-analysis.

No.	Title	Database/Article methodology/Technique used	Sampling/PB Number and MB Number	Nature of sample(s)/PCR genotyping method	Population location	Detection Rate Results	JBI Score*
1	Real-time PCR-based quantitation of viable *Mycobacterium leprae* strain from clinical samples and environmental sources and its genotype in multi-case leprosy families of India (Singh et al., 2020)	PUBMED/ Cross-sectional study/ PCR and RT-qPCR	9 MB cases 16 PB cases	slot skin smear	India	PB (4/16) MB (7/9)	(8/8)
2	Reverse Transcription-PCR Detection of *Mycobacterium leprae* in Clinical Specimens (Kurabachew et al., 1998)	PUBMED/ Case–control study/ RT-PCR	29 MB cases 21 PB cases	Biopsy	Ethiopia	PB (12/18) MB (25/26)	(10/10)
3	Ultra-sensitive detection of *Mycobacterium leprae*: DNA extraction and PCR assays (Manta et al., 2020)	PUBMED/ Cross-sectional study/qPCR	23 MB cases 24 PB cases	Biopsy	Brazil	PB (15/24) MB (23/23)	(7/8)
4	A simplified reverse transcriptase PCR for rapid detection of *Mycobacterium leprae* in skin specimens (Phetsuksiri et al., 2006)	PUBMED/ Cross-sectional study/RT-PCR	36 MB cases 24 PB cases	Slit skin smear	Thailand	PB (13/24) MB (34/36)	(8/8)
5	Evaluation of *16S rRNA* qPCR for detection of *Mycobacterium leprae* DNA in nasal secretion and skin biopsy samples from multibacillary and paucibacillary leprosy cases (Marques et al., 2018)	PUBMED/Case–control study/qPCR	39 MB cases 15 PB cases	Biopsy	Brazil	PB (11/15) MB (25/39)	(10/10)
6	Comparative evaluation of PCR amplification of *RLEP*, *16S rRNA*, *rpoT* and *SodA* gene targets for detection of *M. leprae* DNA from clinical and environmental samples (Turankar et al., 2015)	PUBMED/Case–control Study/PCR	30 MB cases 30 PB cases	Slit skin smear	India	PB (5/30) MB (18/30)	(10/10)

### Results of meta-analysis and publication bias of data

**Figure 2:**
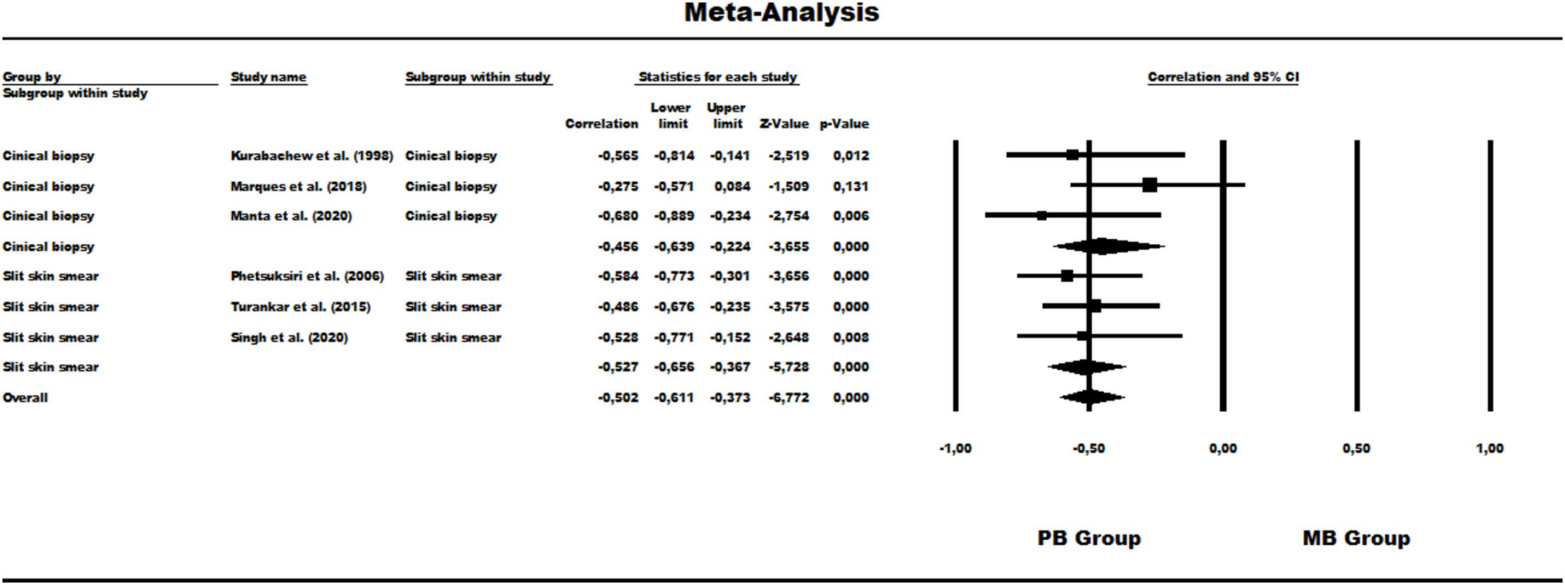
Forest Plot of the correlation of overall leprosy detection about the operational groups of disease classification.

**Figure 3:**
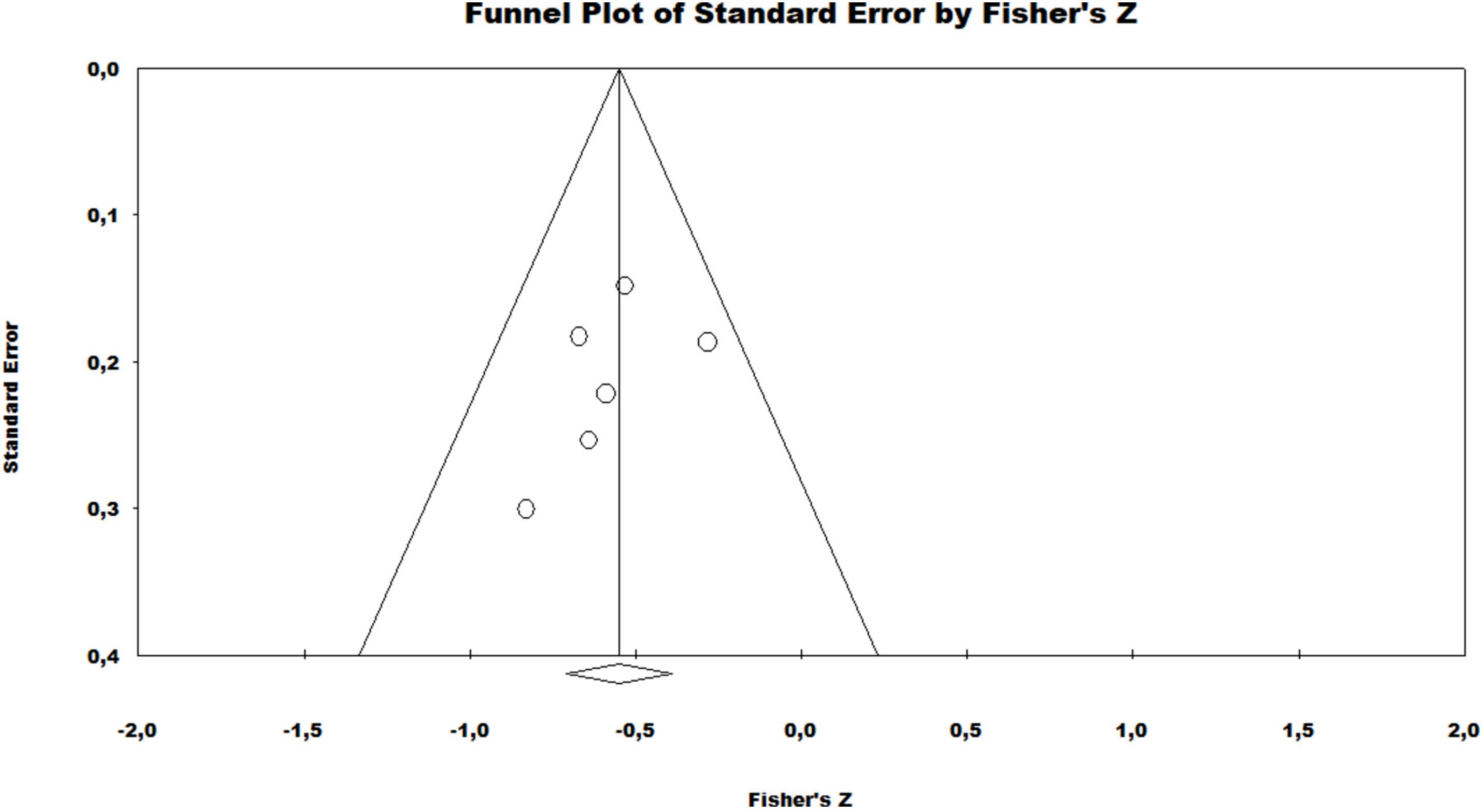
Publication bias assessment funnel plot of the meta-analysis.

The data showed significant variation beyond the null hypothesis point in both subgroup analyses by clinical biopsy and skin smear and also in the total analysis of the data for all sample types, from the perspective of the paucibacillary group (Clinical biopsy: −0.45 [95% CI = −0.63 – –0.22], *p* < 0.001/Skin smear: -0.52 [95% CI = −0.65 – –0.36], *p* < 0.001/Overall: –0.50 [95% CI = −0.61 – –0.37], *p* < 0.001). These data are shown in Figure 2. Therefore, the correlation was pulled towards this group, i.e., there was a prominently more biased detection and, in this case, lower detection of paucibacillary. None of the subgroups or the combined investigation showed high heterogeneity (Clinical biopsy: x2 = 2.85, df = 2, *p* = 0.24, *I*^2^ = 29.86% / Slit-skin smear: x2 = 0.35, df = 2, *p* = 0.84, *I*^2^ = 0%/General: x2 = 3.51, df = 5, *p* = 0.62, *I*^2^ = 0%). With regard to the risk of publication bias, the funnel plot showed no visual disagreement with the data, and Begg’s test showed no statistical significance (*p* = 0.13) (Figure 3).

### Scientometric analyzes

**Figure 4:**
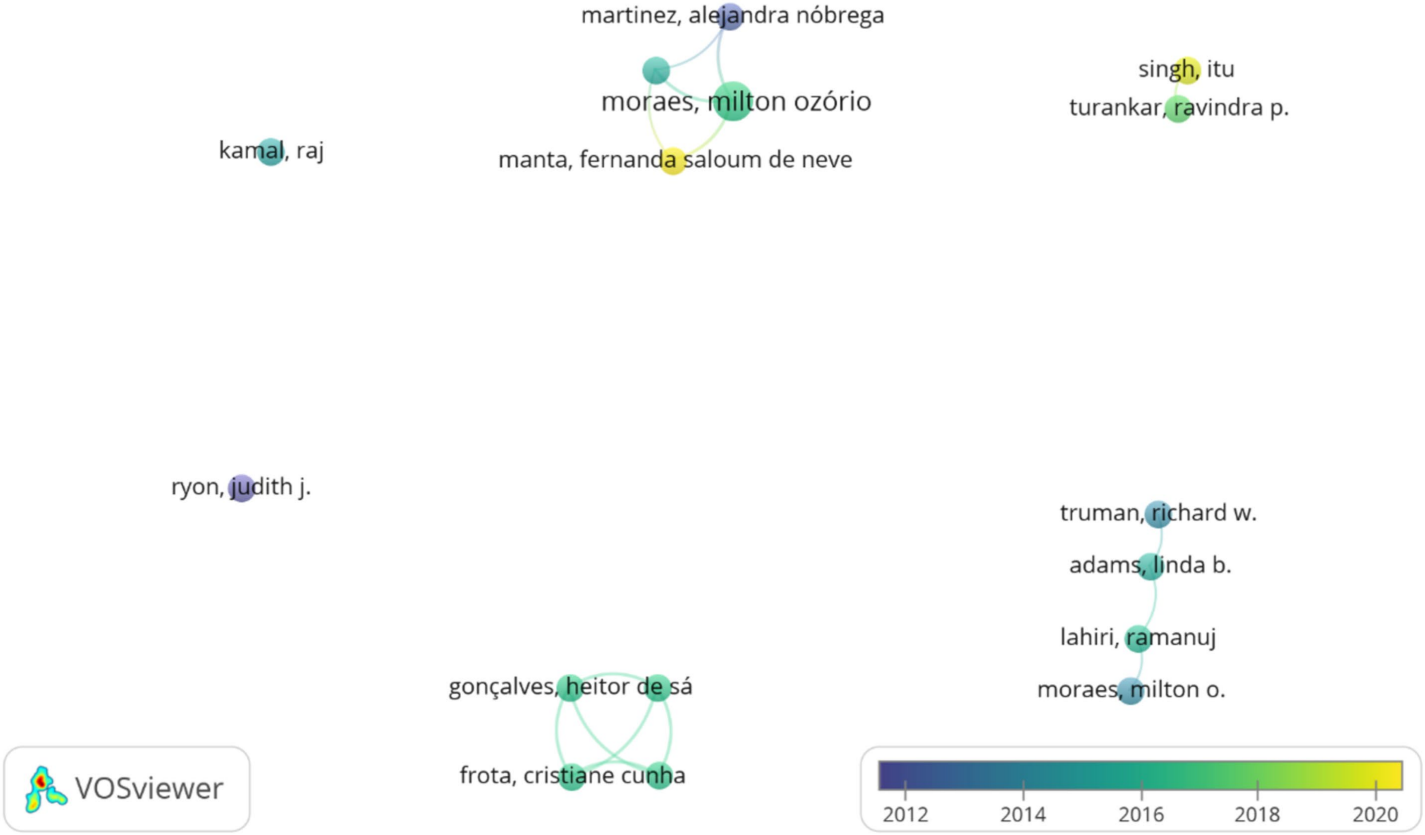
A collaborative network of co-authors in the molecular detection of leprosy (1991–2024). Lighter colors indicate more recent collaborations.

**Figure 5:**
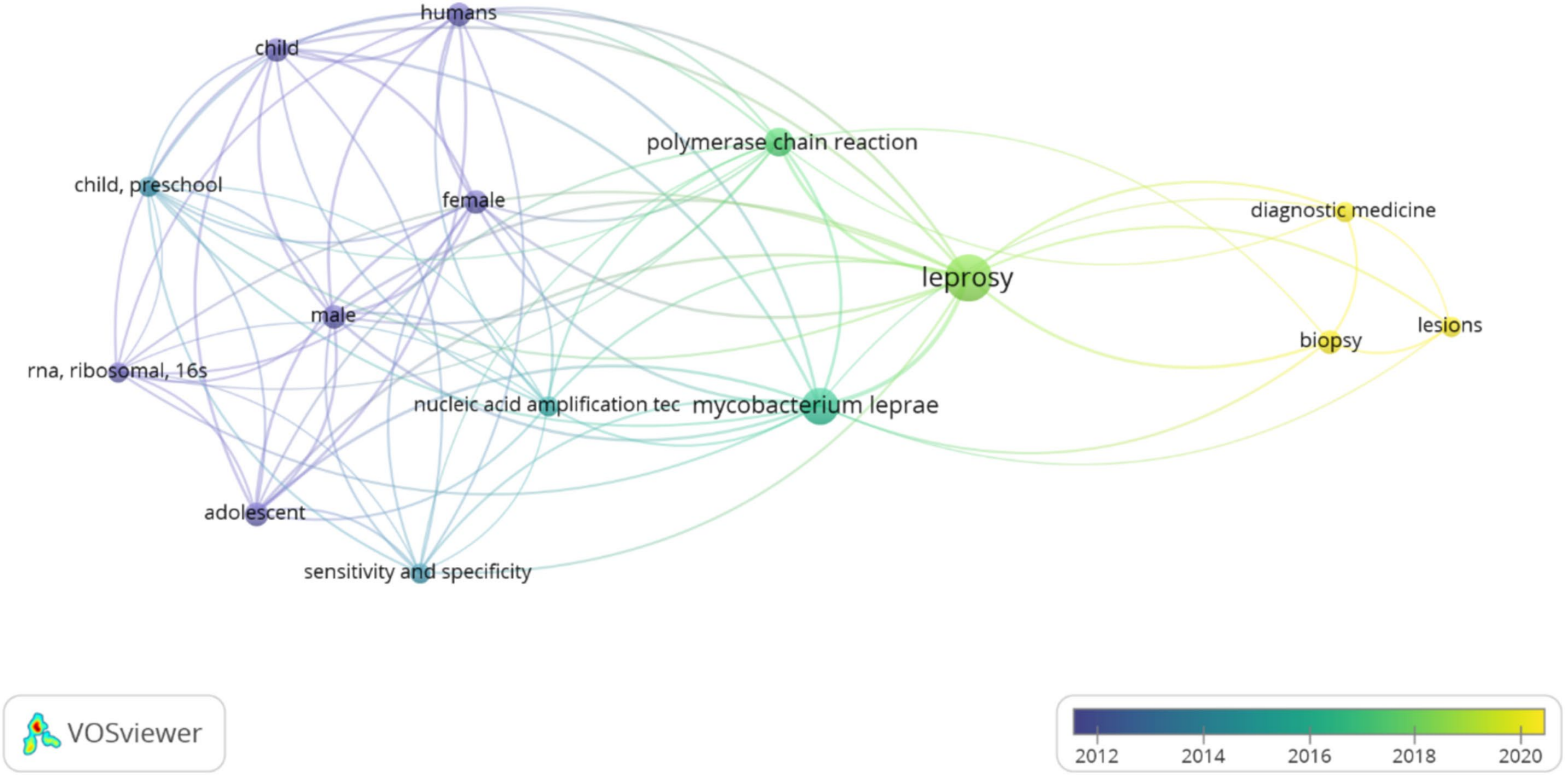
Keyword co-occurrence network about the field of leprosy (1991–2024). Lighter colors indicate more recent use of these terms.

The co-authorship network in the field of leprosy is shown in Figure 4. There were 6 co-authorship clusters. The first, second and third clusters were composed of 4 distinct researchers, the fourth of 2 scholars and the fifth and sixth clusters of only 1 researcher. The size of the circle is proportional to the number of papers published by the author, the color of the circles corresponds to the year of publication, and the thickness of the lines is proportional to the frequency of collaboration. The thickness of the lines between them also testifies to the degree of cooperation, several large collaborative clusters and several smaller ones. As shown in Figures 5, a total of 2 clusters were identified, the first consisting of 9 items and the second of 6 items. The first cluster denoted the following keywords: “adolescent”; “child”; “child, preschool”; “female”; “humans”; “male”; “nucleic acid amplification technique”; “rna, ribosomal, 16S”; “sensitivity and specificity.” The second cluster consisted of: “biopsy”; “diagnostic medicine”; “leprosy”; “lesions”; “*Mycobacterium leprae*”; “Polymerase Chain Reaction.”

**Figure 6:**
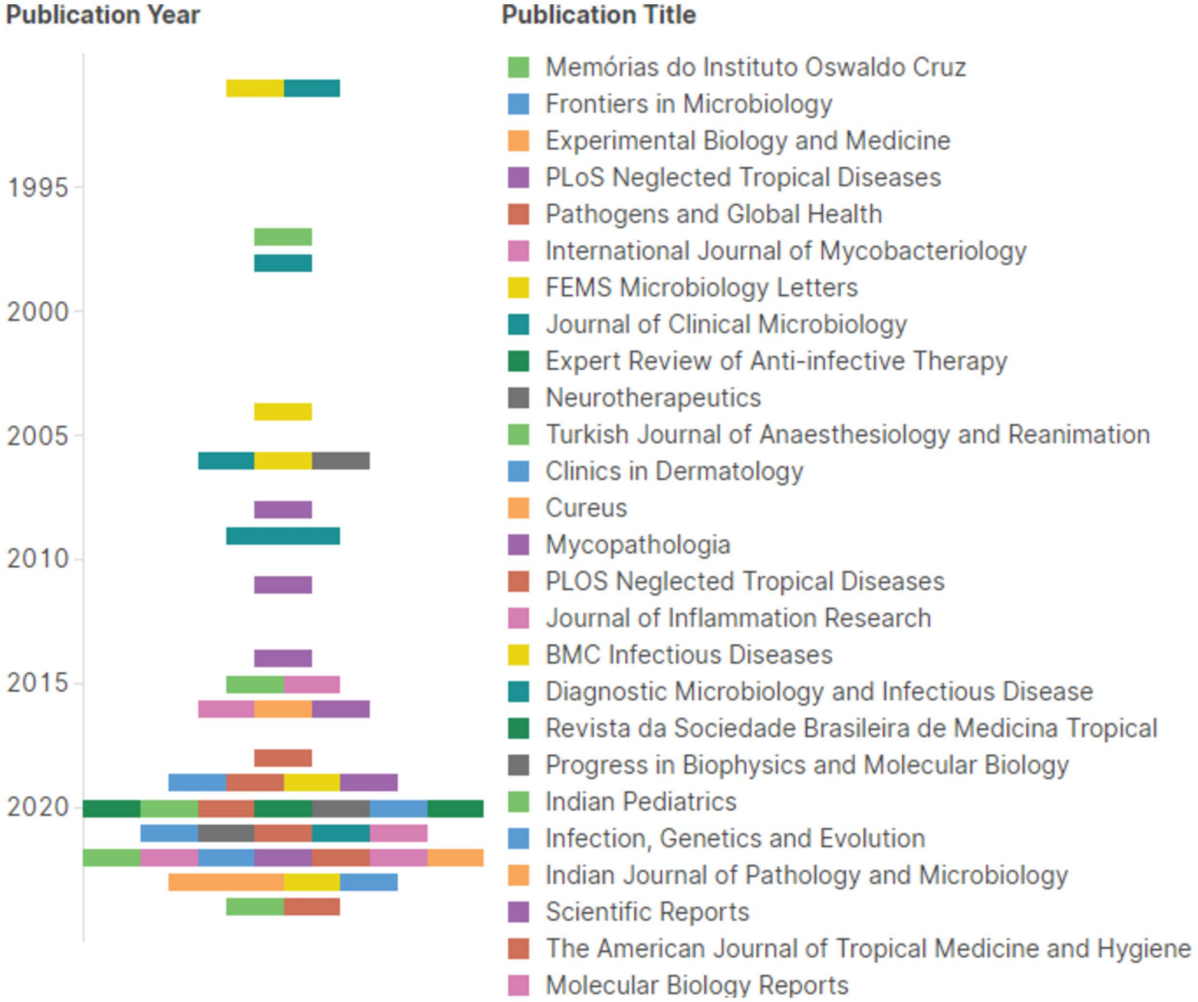
Proportional distribution of studies in relation to the journal in which it was published and the year of publication (1991–2024). Each bracket dot of a different color represents a different periodic.

Figure 6 shows the evolutionary trajectory of the number of papers and journals on this field of leprosy. We obtained 48 papers for this study. The publications increased steadily from 2017 (*n* = 18, 37.5%) to 2024 (*n* = 30, 62.5%). Research on the molecular diagnosis of leprosy was published in 26 distinct journals. “PLOS Neglected Tropical Diseases” (JCR IF = 4.817) was the most productive journal, with 7 related papers (14.58%). It covered leprosy’s medical, physical, diagnosis, treatment, molecular biology, microbiology and social aspects and relevant information on leprosy control, followed closely by “International journal of leprosy and other mycobacterial diseases” which contributed 8.33% to the overall publications. “Journal of Clinical Microbiology” and “Leprosy Review” contributed equal rates of 6.25% of the works. “Journal of Clinical Microbiology” was the highest impact factor of these journals (JCR IF = 6.1), which published 3 papers (6.25%).

## Discussion

Recent studies have demonstrated the superior sensitivity of PCR-based methods over conventional diagnostics. Although *Mycobacterium leprae* belongs to the slow-growing mycobacteria group, its *16S rRNA* gene sequence differs significantly from other slow-growing mycobacteria. A quick and non-radioactive technique for identifying *M. leprae* in infected tissue was developed using these variations (by Polymerase Chain Reaction - PCR) (Cox et al., 1991). For instance, a study evaluating the *16S rRNA* quantitative PCR (qPCR) method reported a positive detection rate of 94% in multibacillary leprosy patients, compared to significantly lower rates in paucibacillary cases (43.8% for nasal swabs and 9.4% for biopsies) (Manta et al., 2020). This discrepancy highlights the need for tailored diagnostic approaches based on the clinical spectrum of leprosy.

The ability to accurately detect *M. leprae* is crucial for timely intervention and management of leprosy. High sensitivity in detecting the bacterium allows for earlier diagnosis, which is essential in preventing transmission and reducing the risk of disabilities associated with the disease. Studies have shown that individuals with positive PCR results are at a higher risk of developing leprosy, highlighting the importance of molecular diagnoses in surveillance and control measures (Avanzi et al., 2020; Malhotra and Husain, 2022).

Regarding the types of diagnosis tests, PCR has been shown to be significantly more sensitive than traditional microscopy for detecting *Mycobacterium leprae*. Microscopy, which relies on the identification of acid-fast bacilli (AFB), requires a minimum bacterial load of approximately 10^4^ organisms per gram of tissue for reliable detection. This threshold often results in missed diagnoses, particularly in paucibacillary forms of leprosy where bacilli are scarce or absent (Siwakoti et al., 2016). In contrast, PCR can detect much lower quantities of bacterial DNA, making it especially valuable for identifying cases that are difficult to diagnose through microscopy alone. However, while PCR offers higher sensitivity, its implementation may be limited by costs and the need for specialized laboratory equipment. Microscopy remains widely used due to its accessibility and lower cost, particularly in resource-limited settings. However, PCR’s ability to provide rapid results can enhance early diagnosis and treatment initiation (Banerjee et al., 2011).

Serological tests, such as enzyme-linked immunosorbent assays (ELISA), have been employed to detect immune responses to *M. leprae* antigens. However, these tests can yield variable results based on the patient’s immune status and the stage of the disease. Studies indicate that serological tests may not be as reliable as PCR in accurately diagnosing leprosy, particularly in cases with low bacillary loads or atypical presentations (Gurung et al., 2019). Although PCR is highly specific and sensitive, it is often recommended to use it in conjunction with serological tests to improve overall diagnostic accuracy. This combined approach can enhance the predictive value of leprosy diagnosis by confirming active infections while also assessing immune responses (Silva et al., 2017).

Molecular methods, particularly PCR targeting the *Mycobacterium leprae* genome, have emerged as superior diagnostic tools for detecting early or latent leprosy cases, especially in patients with PB forms of the disease (Das et al., 2023; David et al., 2024). These advancements hold significant potential for improving clinical outcomes and disease control strategies. In addition to showing higher sensitivity than conventional diagnostic techniques like microscopy and serology, PCR’s speedy identification of *M. leprae* DNA enables earlier diagnosis and treatment commencement. In particular, as PB patients cannot have large antibody responses that can be detected by serological techniques, early management is essential in limiting the course of the disease and its accompanying impairments (Warka et al., 2024).

Furthermore, PCR is applicable to a wide range of biological materials other than skin biopsies, such as blood, urine, and slit skin smears. Through measuring the bacterial load, quantitative polymerase chain reaction, or qPCR, can provide doctors with information on the severity of an illness and possible treatment outcomes. Incorporating qPCR into routine diagnostic protocols can significantly enhance sensitivity. qPCR allows for the quantification of *M. leprae* DNA, making it possible to detect lower bacterial loads effectively. This quantification can lead to better case management by guiding therapeutic decisions and treatment efficacy monitoring over time (Manta et al., 2019).

Only six studies that included molecular research involving the highlighted PCR target gene were included in the present analysis, which differentiates groups based on the operational classification of leprosy. Molecular research that aimed to identify these cases by analyzing patient nasal samples was not found in the search; instead, samples from clinical biopsy or skin smear were the only findings. In this meta-analysis of the clinical spectrum of leprosy, a strong negative correlation was discovered in the correlation for comparative detection analysis. This means that when the detection rate rises in one group, it falls in the other group for both clinical biopsy samples and skin smears from leprosy patients. This might suggest that this gene has a high specificity but a low sensitivity as a detection target.

In the context of leprosy diagnostics, the trade-off between sensitivity and specificity in PCR assays is a critical consideration, particularly when evaluating the performance of different markers like the *16S rRNA* gene and *M. leprae*-specific repetitive element (*RLEP*). High sensitivity is crucial for detecting active infections, particularly in PB cases where bacterial loads are low. A sensitive test minimizes the risk of false negatives, which can lead to untreated infections and ongoing transmission. The inability to accurately detect *M. leprae* DNA in PB cases can lead to delayed diagnosis and treatment initiation, which is particularly concerning as early intervention is crucial for preventing nerve damage and disability (Van Veen et al., 2006; Marques et al., 2018). High specificity is important to avoid false positives, which can cause unnecessary anxiety and lead to inappropriate treatment strategies. However, in clinical practice, especially in endemic regions, the priority often shifts towards sensitivity when diagnosing diseases with low prevalence or variable presentation (Martins et al., 2016).

The differing detection rates of *Mycobacterium leprae* in skin biopsies and nasal swabs can be attributed to several factors, including bacterial localization in various tissue types and methodological differences in sample preparation (Sarath et al., 2023). Understanding these variations is essential for addressing the diagnostic challenges faced in clinical practice. *M. leprae* exhibits a preference for specific tissues, particularly those with cooler temperatures, such as the skin and peripheral nerves. In skin biopsies, the bacterium is often found in higher concentrations due to its localization in dermal tissues, where it can cause lesions (Kramme et al., 2004). This difference in bacterial load directly affects the sensitivity of PCR assays; higher concentrations of *M. leprae* in skin biopsies increase the likelihood of successful DNA amplification (Santos et al., 1993).

The clinical spectrum of leprosy also influences bacterial distribution. In MB cases, where there is a higher overall bacterial load, both skin and nasal samples are more likely to yield positive results. However, in PB cases, where bacillary counts are low, nasal swabs may not capture sufficient *M. leprae* DNA for detection. Therefore, for PB cases, clinicians may need to prioritize skin biopsies or other invasive techniques to ensure adequate bacterial recovery for accurate diagnosis. For surveillance, nasal swabs offer a non-invasive alternative but may require confirmation through more sensitive methods or additional sampling strategies (Job et al., 1991; De Wit et al., 1993).

This implies the possible invalidation of this PCR method with this target for complementary diagnosis of paucibacillary leprosy, as it is not very effective for use in detecting the disease in this operational group. This is corroborated by previous data from molecular research involving leprosy diagnosis. PCR using the *16S rRNA* gene as a target has lower sensitivity in detecting *M. leprae* when compared to other genomic regions, such as the *M. lepraerlep* sequence (Kamal et al., 2006; Martinez et al., 2011; Turankar et al., 2015; Marques et al., 2018; Sharma et al., 2024). Furthermore, this possible high specificity should be further investigated, considering that this finding may be related solely to the high bacillary load in patients and, therefore, to the greater number of copies and viability of *M. leprae* in the samples available in each study of this present review (Kurabachew et al., 1998; Xiong et al., 2006; Truman et al., 2008).

In particular, Martinez et al. (2009) confirmed the specificity of the *16S rRNA* primer for *M. leprae* using the qPCR technique on 9 other Mycobacterium species, including *M. tuberculosis* H37RVV ATCC 27294 (Martinez et al., 2009). Study carried out by Martinez et al. (2011), comparing the sensitivity and specificity of qPCR in the amplification of the *sodA*, *16S rRNA*, *RLEP* and *Ag85B* genes for the differential diagnosis of leprosy, referred to *RLEP* as a target with greater sensitivity for leprosy cases. About *16S rRNA*, this was more specific, although less sensitive (Martinez et al., 2011). *Ag85B* and *sodA* have been shown to have high sensitivity in detecting *M. leprae* but they are not as widely adopted as *16S rRNA* due to variability in their performance across different studies and sample types (Torres et al., 2021). The *Ag85B* gene is associated with virulence but may not provide as clear a differentiation between active disease and latent infection as *16S rRNA* when used alone (Spencer and Brennan, 2011; Spencer et al., 2011).

The 18 kDa protein is a major antigen of *M. leprae* and plays a crucial role in the immune response. Specific primers targeting this gene have shown high specificity in detecting *M. leprae* DNA. While effective, its sensitivity can be variable, particularly in paucibacillary forms of leprosy where bacterial load is low (Kang et al., 2003; Machado et al., 2020). Besides that, multidrug therapy (MDT)’s effectiveness in treating leprosy may be effectively evaluated using RT-PCR and DNA-PCR for the 18 kDa protein of *M. leprae* (Chae et al., 2002). The 65 kDa protein is a heat shock protein that aids in the survival of *M. leprae* under stress conditions. This gene has been extensively studied for its role in PCR diagnostics, often used alongside other targets to enhance detection rates. Regarding its performance, while it provides good specificity, its sensitivity can be low, particularly in low-bacterial-load cases (Hartskeerl et al., 1989; Donoghue et al., 2001; Cheng et al., 2019). The 36 kDa is associated with the virulence of *M. leprae* and is another significant component recognized by the immune system. PCR assays using this target have been reported to provide reliable results, especially in cases with atypical presentations or when other methods fail to confirm leprosy. The 36 kDa antigen has demonstrated good performance in terms of both sensitivity and specificity, making it a preferred choice for molecular diagnostics (Wichitwechkarn et al., 1995; Shampa et al., 2020).

The *16S rRNA* gene is a single-copy target in the *M. leprae* genome, making it highly specific but potentially less sensitive than multi-copy targets like *RLEP*. *16S rRNA* PCR can detect viable bacilli by targeting ribosomal RNA, which is only present in metabolically active organisms. One study found that *16S rRNA* qPCR had a sensitivity of only 20% in slit skin smears (SSS) from household contacts, likely due to the low bacterial loads in these samples (Manta et al., 2019).

The *RLEP* sequence is a repetitive element with multiple copies (at least 28) in the *M. leprae* genome, conferring higher sensitivity compared to single-copy targets like *16S rRNA*. *RLEP* qPCR demonstrated 100% sensitivity in detecting *M. leprae* DNA in nasal swabs from MB leprosy patients, but it cannot distinguish between viable and non-viable organisms or between active disease and latent infection. In summary, *RLEP* PCR is ideal for rapid, sensitive detection of *M. leprae* in active disease, particularly MB cases. *RLEP* is also highly conserved among other mycobacterial species, which raises concerns about potential cross-reactivity and false positives in certain contexts. *16S rRNA* PCR provides valuable information about bacterial viability and infectivity, making it useful for monitoring treatment response and assessing subclinical infections. Given its limitations, it would be preferable for *16S rRNA* PCR to be frequently complemented by other diagnostic methods, such as *RLEP* PCR or serological tests, to improve the overall accuracy of the diagnosis of leprosy patients, especially those with PB forms (Teske et al., 1991; Yan et al., 2014; Mohanty et al., 2020).

Furthermore, the operational classification of leprosy patients (multibacillary vs. paucibacillary) influences the detection rates. In a comparative analysis, multibacillary patients demonstrated a markedly higher positivity in PCR tests, suggesting that the clinical spectrum must be considered when interpreting PCR results and planning treatment strategies (Marques et al., 2018). Furthermore, using different sample types has been shown to affect detection rates. Skin biopsies and nasal swabs yielded higher positivity rates than peripheral blood, particularly in multibacillary cases (Manta et al., 2019). The choice of extraction methods also plays a critical role; for example, the Microbiome kit significantly enhances the detection of *M. leprae* DNA, indicating that optimized sample preparation can improve diagnostic accuracy (Lopes-Luz et al., 2023).

Moreover, scientometric analysis could provide valuable insights into the evolution of leprosy research and the effectiveness of diagnostic techniques. The scientometric analysis revealed some scientific cooperation between authors and a large body of literary works produced in the last 8 years. The increase in the number of publications related to the molecular diagnosis of leprosy in the last 8 years can be justified by several factors, mainly by the advances in molecular biology and its dissemination in public health. Improving molecular biology techniques, such as PCR, has enabled more sensitive and specific detection of *Mycobacterium leprae* (Martinez et al., 2014). Recent studies have shown that molecular methods offer a more reliable alternative than traditional diagnostic methods, which often have low sensitivity, especially in the early stages of the disease (Martinez et al., 2011; Marques et al., 2018; Torres et al., 2021). Leprosy continues to be a significant concern in several regions of the world, and organizations such as the WHO have promoted initiatives to control and eliminate leprosy, encouraging research in molecular diagnostics (Santos et al., 2020). These factors, combined with institutional support and research collaboration, have boosted scientific production in this area.

Despite advances in PCR technology, challenges remain in the widespread implementation of these methods. Issues such as the need for specialized laboratory infrastructure, the potential for contamination, and the variability in sensitivity based on clinical presentation complicate the diagnostic landscape (Lima et al., 2015). Addressing the variability in sample collection methods and PCR techniques is essential for improving the robustness and reproducibility of molecular diagnostics research for leprosy. The PCR target selection and the technique used for sample collection (e.g., punch biopsy vs. excisional biopsy) can affect the quality and quantity of the sample obtained. Inconsistent methods may result in varying amounts of viable bacteria or DNA, impacting PCR sensitivity and specificity. Through establishing standardized protocols, providing training, implementing quality control measures, encouraging data sharing, and regularly reviewing methodologies, researchers can enhance the comparability of results across studies. These efforts can ultimately contribute to more reliable diagnostic tools that can effectively support leprosy management and control strategies globally. For instance, multiplex PCR has shown promise in detecting early leprosy cases (Pathak et al., 2024).

Then, multiplex PCR allows for the simultaneous amplification of multiple DNA targets within a single reaction. This capability is particularly beneficial in leprosy diagnostics, where it can include both *RLEP* and *16S rRNA* genes, among others. By targeting multiple sequences, multiplex PCR increases the likelihood of detecting *M. leprae* DNA even when bacterial loads are low. Furthermore, low-bacterial-load samples may contain inhibitors that affect PCR performance (Bertão-Santos et al., 2024). Multiplex assays can be designed to include internal controls that help identify and mitigate these inhibitory effects, ensuring more reliable results. Through optimization of reaction conditions and using specific primers designed to minimize non-specific amplification, multiplex PCR can enhance overall assay performance. Research indicates that multiplex PCR can significantly improve diagnostic sensitivity in smear-negative samples, which is particularly relevant for PB cases (Banerjee et al., 2010).

Additionally, the integration of molecular diagnostics into routine clinical practice faces barriers, including cost, training requirements, and the availability of reagents. Establishing standardized protocols and training programs is essential to enhance the reliability of PCR-based diagnostics in diverse healthcare settings (Barreto Da Silveira et al., 2021). Although *16S rRNA* is widely used as a complementary diagnostic test for leprosy, clinical variability and low *M. leprae* load in paucibacillary cases may result in inaccurate diagnoses (Kampirapap et al., 1997; Collins et al., 2023). This suggests the need to improve molecular methods, such as qPCR, which demonstrate greater sensitivity and specificity, to improve the detection and diagnosis of leprosy ultimately.

More advanced instruments are needed to demonstrate viability since *M. leprae* cannot be grown on artificial medium. A few studies have shown that RNA assays may be applied to evaluate bacterial viability under MDT in Slit Skin Smear and skin biopsies. *M. leprae* RNA detection is thought to be a feasible option to identify viable/replicating organisms (Lini et al., 2009; Martinez et al., 2009, 2014). Viable *M. leprae* has been found in environmental samples taken from the local area surrounding the homes of leprosy patients, which has led to the increased use of RNA tests in transmission investigations (Mohanty et al., 2016; Turankar et al., 2016).

Martinez et al. (2009) have previously demonstrated that the low sensitivity of *M. leprae* mRNA (such as *sodA*) in clinical samples limits its use to short-term experimental conditions for predicting the survivability of the bacilli (Martinez et al., 2009). Other (myco-) bacterial pathogens were also found to have low mRNA detection sensitivity from clinical samples [32], however, some authors contended that rRNA, despite its high sensitivity, could also be detected from dead bacteria (including metabolically active but culture-negative bacilli such as those of the *Mycobacterium tuberculosis* complex—MTBC). Recent research by Prakoeswa et al. (2016) confirmed the results of a previous study by Haile and Ryon (2004), showing that *16S rRNA* is quickly degraded in dead *M. leprae* and may thus be utilized as a viability marker (Haile and Ryon, 2004; Prakoeswa et al., 2016).

The advantages of implementing the *16S rRNA* technique in diagnosing leprosy are the robustness in routine screening; clinical validation (*16S rRNA* PCR has shown reasonable sensitivity [around 50%] and high specificity [approximately 94%] when used on skin biopsies from suspected leprosy patients) (Manta et al., 2020); complementary role in the case of integration of *16S rRNA* PCR with other diagnostic methods (such as *RLEP* or serological tests) can enhance overall diagnostic accuracy (Bathula et al., 2023). Through the use of multiple targets, clinicians can improve sensitivity while maintaining specificity, thereby reducing the likelihood of false negatives. Besides that, ongoing research into optimizing *16S rRNA*-based assays—such as developing multiplex PCR that includes both *RLEP* and *16S rRNA*—can further enhance its utility in clinical practice (Beissner et al., 2019).

The limitations of this review permeate the heterogeneity of the studies included, as the variability in sample collection methodologies, types of PCR used, and patient inclusion criteria between the studies analyzed can lead to inconsistent results. Studies using different DNA extraction techniques or molecular targets may not be directly comparable, influencing the reported detection rate. The sensitivity and specificity limits of the technique may influence the reported comparison. Furthermore, the representativeness of the samples is crucial, where sampling must be respected for causal inference, and unequal sample groupings (between paucibacillary and multibacillary) can distort conclusions about the effectiveness of PCR in different clinical spectrums (Margotti and de Paiva, 2021).

The distribution of leprosy cases may be greatly impacted by variables including population density, socioeconomic level, and healthcare facilities, which can have an impact on both detection and categorization. Increased poverty levels are frequently associated with worse health outcomes, including increased leprosy prevalence (Nery et al., 2019). A lack of knowledge about leprosy symptoms and transmission caused by lower educational level might delay diagnosis and treatment (Alves et al., 2014). In impoverished areas, educational gaps are frequently more noticeable. In economically poor communities, there is a higher prevalence of inadequate housing and sanitation, which contributes to the development of infectious illnesses like leprosy (Prakoeswa et al., 2020).

Rural and underdeveloped communities frequently have restricted access to healthcare services. Less developed areas could find it difficult to diagnose and treat leprosy cases promptly, which could impact whether instances are classified as PB or MB. Furthermore, effective disease monitoring and response depend on a strong public health infrastructure and public health interventions, meaning that national and local health policies that emphasize leprosy control can have a major impact on prevalence rates (Mártires et al., 2024). Geographic heterogeneity is a critical factor influencing leprosy detection rates. Regions with high incidence rates of leprosy, such as parts of India and Brazil, may have a higher proportion of MB cases due to ongoing transmission of the bacillus. In contrast, areas with low incidence may have more PB cases, where the infection is often detected at an early stage (Paz et al., 2023). In Brazil, for example, regions like Pará exhibit significantly higher new case detection rates compared to other states due to ongoing transmission dynamics and historical factors related to healthcare access (Dergan et al., 2023; Queiroz et al., 2024). This geographic variability can lead to differences in reported heterogeneity in this present meta-analysis.

The clinical spectrum of leprosy includes various stages, from asymptomatic infections to severe forms with significant disabilities. The stage at which patients are diagnosed can influence detection rates and outcomes. For example, studies that mainly include patients with advanced disease may record higher detection rates due to the more severe presentation of symptoms. On the other hand, studies that focus on early detection and asymptomatic cases may show lower rates (Mahato et al., 2023). Therefore, this may also be a limitation of our analyses.

Variations in study design also contribute to heterogeneity in findings. Differences may arise from: methodological approaches some studies may employ different diagnostic criteria or methodologies [e.g., varying sample sizes], leading to discrepancies in detection rates; population characteristics (age, sex, ethnicity) of study populations can influence disease prevalence and reporting; data collection methods (studies utilizing retrospective data might capture different aspects of disease prevalence compared to prospective studies and this can lead to biases in how cases are reported and detected) (Romão and Mazzoni, 2013; Freitas et al., 2017; Albuquerque et al., 2020; Barbosa et al., 2020).

Several approaches are being considered to overcome these limitations in the molecular technique on leprosy. The combination of different molecular targets, such as the proposal of a combined *RLEP*/*16S rRNA* assay for detecting *M. leprae* from nasal swab samples, is one of the solutions already discussed in the literature. The multi-target use of a molecular technique combining *RLEP* and *16S rRNA* in a real-time quantitative PCR (qPCR) assay takes advantage of the strengths of both markers: *RLEP*, which increases sensitivity for the detection of low bacillary loads; *16S rRNA*, which can provide valuable information on bacterial viability (Turankar et al., 2015). This approach aims to increase the sensitivity of the diagnosis, especially in paucibacillary cases, where the bacterial load is low and traditional methods often fail. Therefore, this method is helpful about the issue of bacillary load and viability of *M. leprae* from nasal swab samples (Beissner et al., 2019). It can also be used for early diagnosis, tracking the effectiveness of treatment, and examining the potential role of *M. leprae*’s nasal carriage in aerosol infection-mediated human-to-human transmission.

Recent research has explored the efficacy of different PCR methods for detecting *M. leprae*. For example, ultra-sensitive detection of *M. leprae* using DNA extraction methods and PCR assays has shown promising results, with high detection rates in biopsy specimens (Manta et al., 2020). Another study highlighted the use of RT-PCR for detecting *M. leprae* in clinical specimens, showing high specificity, especially in multibacillary cases (Kurabachew et al., 1998). Then, it is crucial to continue exploring and validating new methodologies that can overcome the current limitations in detecting *M. leprae*. Combining different molecular approaches and improving diagnostic techniques can increase the detection rate and contribute to more effective leprosy control in affected populations. Collaboration between researchers, clinicians, and healthcare institutions will be essential to implement these innovations and improve patient outcomes (Arthaningsih and Margha, 2023).

Improving molecular diagnostics for leprosy, particularly in PB cases, is crucial for early detection, timely treatment, and effective disease control. Using multiple gene targets (e.g., *RLEP* and *16S rRNA*) in a multiplex PCR assay can enhance overall sensitivity (Beissner et al., 2019). This approach increases the likelihood of detecting *M. leprae* DNA across different clinical presentations and sample types. Combining PCR with immune-based diagnostics, such as IFN-*γ* release assays (IGRAs), can provide a holistic view of the disease state (Kim et al., 2013; Barreto Da Silveira et al., 2021). While PCR confirms the presence of the pathogen, IGRAs assess the host’s immune response to specific antigens from *M. leprae*. This dual approach can differentiate between active infections and latent states, improving clinical decision-making. Focus on areas with higher bacterial loads when collecting skin samples (e.g., earlobes or lesions) can increase the chances of detecting *M. leprae* (Lini et al., 2009). Ensure that sample processing protocols are standardized to maximize DNA recovery and minimize degradation. This includes optimizing extraction techniques to enhance yield from low-bacterial-load samples. Moreover, it can be considered to use DNA extraction kits specifically designed for low-yield samples, which can improve the quality and quantity of extracted DNA (Soto and Torres Muñoz, 2015).

The integration of RNA-based viability assays and the exploration of emerging technologies like Next-Generation Sequencing (NGS) and CRISPR-based diagnostics hold promise for enhancing sensitivity and specificity. RNA targets are more abundant than DNA in actively replicating bacteria, allowing for the detection of viable organisms even when bacterial loads are low, as often seen in PB cases. Through quantifying RNA levels, clinicians can monitor treatment efficacy and identify potential treatment failures early on. A study found that a combined *RLEP* DNA/*16S rRNA* assay could consistently detect viable *M. leprae* in MB patient biopsies before treatment and demonstrate a decline in viability during multidrug therapy (MDT) (Donoghue et al., 2001). RNA-based assays can be applied to environmental samples to study the role of nasal carriage in human-to-human transmission, as viable bacteria can be detected through RNA markers (Fischer, 2017).

NGS technology allows for the simultaneous detection of multiple genetic targets, potentially improving sensitivity compared to single-target PCR assays. NGS can also provide insights into *M. leprae* strain diversity and its association with clinical outcomes (Quan et al., 2020). CRISPR-Cas systems have shown potential for rapid, sensitive, and specific detection of pathogens. Through targeting conserved regions in the *M. leprae* genome, CRISPR-based assays could achieve high sensitivity while maintaining specificity. As research continues to validate these methods, their clinical adoption could significantly impact leprosy management and contribute to the global effort towards disease elimination (Singh et al., 2015). Investigations into new genetic targets or biomarkers that could be better able to identify *M. leprae* at low concentrations or in the early stages of infection could be supported. Clinical trials that assess novel diagnostic instruments and techniques created especially for PB leprosy could be supported.

## Conclusion

The comparative analysis of leprosy detection rates through PCR targeting the *16S rRNA* gene reveals significant insights into the disease’s clinical spectrum and diagnostic challenges. While PCR offers a more sensitive and specific diagnostic tool, its implementation must be carefully managed to address the existing challenges. One way to increase the chances of detecting *M. leprae* DNA in a variety of clinical presentations and sample types is to use alternative approaches, such as combining PCR with immune-based diagnostics, searching for new molecular targets for use in PCR, such as possibly the 36 kDa antigen, or future research directions that combine multiple molecular targets, such as *RLEP* and *16S rRNA* in a multiplex PCR assay. Future research should focus on optimizing diagnostic protocols, exploring less invasive sampling techniques, and enhancing the accessibility of molecular diagnostics in endemic regions. This approach will ultimately contribute to more effective leprosy control and management strategies.

## Data Availability

The original contributions presented in the study are included in the article/supplementary material, further inquiries can be directed to the corresponding author.

## Footnotes

^1^https://www.graphica.app/
